# A Facile Way to Prepare Hydrophilic Homogeneous PES Hollow Fiber Membrane via Non-Solvent Assisted Reverse Thermally Induced Phase Separation (RTIPS) Method

**DOI:** 10.3390/polym11020269

**Published:** 2019-02-05

**Authors:** Min Liu, Anne Ladegaard Skov, Sheng-Hui Liu, Li-Yun Yu, Zhen-liang Xu

**Affiliations:** 1 Key Laboratory for Ultrafine Materials of Ministry of Education, Shanghai Key Laboratory of Advanced Polymeric Materials, School of Materials Science and Engineering, East China University of Science and Technology (ECUST), 130 Meilong Road, Shanghai 200237, China; 2 Danish Polymer Center, Department of Chemical and Biochemical Engineering, Technical University of Denmark, Building 227, 2800 Kgs. Lyngby, Denmark; al@kt.dtu.dk (A.L.S.); lyyu@kt.dtu.dk (L.-Y.Y.); 3 State Key Laboratory of Chemical Engineering, Membrane Science and Engineering R&D Lab, Chemical Engineering Research Center, East China University of Science and Technology (ECUST), 130 Meilong Road, Shanghai 200237, China; lshlsh0562@163.com

**Keywords:** polyethersulfone, sulfonated polyethersulfone, membrane, reverse thermally induced phase separation

## Abstract

Sulfonated polyethersulfone (SPES) was used as an additive to prepare hydrophilic poly(ethersulfone) (PES) hollow fiber membranes via non-solvent assisted reverse thermally induced phase separation (RTIPS) process. The PES/SPES/*N*,*N*-dimethylacetamide (DMAc)/ polyethylene glycol 200 (PEG200) casting solutions are lower critical solution temperature (LCST) membrane forming systems. The LCST and phase separation rate increased with the increase of SPES concentrations, while the casting solutions showed shear thinning. When the membrane forming temperature was higher than the LCST, membrane formation mechanism was controlled by non-solvent assisted RTIPS process and the also membranes presented a more porous structure on the surface and a bi-continuous structure on the cross section. The membranes prepared by applying SPES present higher pure water flux than that of the pure PES membrane. The advantages of the SPES additive are reflected by the relatively high flux, good hydrophilicity and excellent mechanical properties at 0.5 wt.% SPES content.

## 1. Introduction

Polyethersulfone (PES) is a thermoplastic polymer developed by ICI Company in 1972. It has excellent chemical resistance, good thermal stability and excellent mechanical properties, and it has been widely used for membrane materials [1,2,3,4]. At the same time, because of its excellent biocompatibility, PES has gradually been paid attention to by the medical community. Especially in the field of blood purification, it can be utilized as dialysate membrane and plasma separation membrane [5,6,7] and has a broad prospect of development. However, in its pristine form, PES has a serious disadvantage, namely its poor hydrophilicity. When it is used as a membrane material, the poor hydrophilicity makes the membrane fouling and is not easy to clean, thus reducing the separation performance and service life of the membrane [8], which hinders its application in many fields.

At present, the main methods to improve the hydrophilicity of PES membranes include matrix modification and surface modification [9], such as blending [10,11], grafting [12], surface impregnation coating [13], surface chemical modification [14], and irradiation surface grafting [15,16]. Of these methods, the blending modification not only maintains the physical and mechanical properties of the PES, but also improves the hydrophilic property of PES, the water permeability and the fouling resistance of the membrane, and also improves the blood compatibility of the PES. It is a simple and effective way to improve hydrophilicity of PES membrane. Many investigations have successfully prepared hydrophilic PES membranes by blending hydrophilic material with PES [17,18,19,20,21,22,23,24]. Heru et al. [18] prepared PES ultrafiltration membranes by NIPS method using polyvinylpyrrolidone (PVP), poly(ethylene glycol) (PEG) and poly(ethylene oxide)-b-poly(propylene oxide)-b-poly(ethylene oxide) (Pluronic) as macromolecular additives. Their results showed that Pluronic possesses the best behavior across all properties. Han et al. [21] prepared Mg(OH)_2_/PES hybrid membranes by in-suit synthesized phase inversion method, and the results showed that nano-sized Mg(OH)_2_ was formed and distributed uniformly in PES matrix membrane. With the increase of Mg(OH)_2_ content, the membrane’s hydrophilicity, porosity, and permeation flux improved. This research provided a new line for the preparation of hydrophilicity homogeneous hybrid membranes. In the studies of Ahmed et al. [24], graphene oxide (GO) was combined with pore former (PVP, reverse triblock Pluronic, and poloxamine Tetronic (T904)) for the preparation of PES ultrafiltration membranes. The addition of pore formers resulted in synergistic effects with GO. This research indicates that GO, as a performance-enhancing additive for the preparation of hydrophilic PES membrane, is promising. These modifications, however, are based on the non-solvent induced phase separation (NIPS) membrane formation technology, and they result in complicated composition of membrane forming system and membrane forming process, which lead to high cost of production.

As a new membrane formation technology, our earlier studies [25,26,27,28,29,30] introduced a novel reverse thermally induced phase separation (RTIPS) technology. The RTIPS method combines a low membrane formation temperature with fast heat transfer. In the RTIPS process, a lower critical solution temperature (LCST) system is used to prepare membranes. The phase separation driving force for RTIPS is fast heat transfer. Compared to the NIPS method, there are fewer variables to control, and the prepared polymer membranes have a porous top surface and bi-continuous morphology, which usually leads to a high pure water flux and good mechanical properties. Meanwhile, based on the excellent physicochemical properties of sulfonated polyethersulfone (SPES) [31,32,33], SPES was chosen as the hydrophilic material for the PES membrane preparation. SPES not only has similar molecular structure to PES, but also has strong hydrophilicity. Due to the relatively high price, it is necessary to prepare hydrophilic PES-base membrane by combining the advantages of PES and SPES. However, the membrane formation system is a LCST system, which is different from that of the upper critical solution temperature (UCST) system of TIPS. The change of hydrogen bonding interaction in the membrane forming system is the key factor affecting membrane formation mechanism during the RTIPS process. The addition of hydrophilic SPES is connected to induced changes in hydrogen bonding interactions. There are no previous studies about the effects of SPES on the phase separation mechanism, membrane morphology, and performance via non-solvent assisted RTIPS.

In this research, therefore, the PES/SPES/solvent/non-solvent systems with LCST were examined. SPES was used as hydrophilic material, DMAc was used as a good solvent, and polyethylene glycol (PEG) with molecular weight of 200 was used as non-solvents. Hydrophilic PES-based hollow fiber membranes were prepared by non-solvent assisted RTIPS method. Moreover, the effects of SPES concentration and membrane formation temperature on the RTIPS phase separation process, membrane morphology, permeability, hydrophilicity, thermal and mechanical properties are investigated in detail.

## 2. Materials and Methods 

### 2.1. Materials

Polyethersulfones (PES) (M_w_ = 51,000 g/mol) was supplied by BASF Co. Ltd., (Ludwigshafen, Germany). Sulfonated polyethersulfone (SPES) (M_w_ = 40,000 g/mol, degree of sulfonation is 5%) was obtained from Kete Chemicals Co. Ltd. (Changzhou, China). PES and SPES were dried for 24 h at 60oC before use. *N*,*N*-dimethylacetamide (DMAc), polyethylene glycol with molecular weight of 200 (PEG200) and glycerol were purchased from Shanghai Chemical Reagent Co. Ltd. (Shanghai, China). Bovine serum albumin (BSA, M_w_ = 67,000 g/mol), which was used to investigate the hydrophilic properties, was obtained from Shanghai Lianguan Biochemical Engineering Co., Ltd. (Shanghai, China). All chemicals were used as received and without further purification.

### 2.2. Preparation of the Casting Solutions

The compositions of the PES/SPES/solvent/non-solvent casting solutions are shown in Table 1. DMAc is a good solvent for PES and SPES, and PEG200 is poor solvent for PES and SPES. Homogeneous casting solutions were obtained by stirring for 48 h at room temperature, and after that the solutions were degassed under atmospheric pressure for 24 h at room temperature. 

### 2.3. Characterization of the Casting Solutions

#### 2.3.1. Viscosity

The viscosities of the casting solutions with different SPES concentrations were investigated by a DV-Ⅱ+PRO Digital Viscometer (Brookfield, Middleboro, MA, USA) at 25 °C controlled by a constant temperature water bath.

#### 2.3.2. Cloud Point

The cloud point (T_c_), which was referred to as the phase separation temperature of the homogeneous casting solution (i.e., LCST), was measured as described by Liu et al. [30] At first, transparent homogeneous casting solution was placed into a glass tube, and then heated slowly in a water bath. When the phase separation occurred, the onset of turbidity was considered as an indication of the cloud point. 

#### 2.3.3. Light Transmittance Measurement

To investigate the effects of SPES concentration on phase separation kinetics during membrane-forming process, light transmittance measurements were carried out [26]. The light intensity indicates the phase separation rate of the PES/SPES/DMAc/PEG200 casting solution, and the intensity of the light through the casting solution was recorded as a function of time.

### 2.4. Preparation of Hollow Fiber Membranes

The hollow fiber membranes were prepared by non-solvent assisted RTIPS spinning method. During the membrane spinning process, deionized water was used for the internal and external coagulation baths, the temperatures of which are shown in Table 2, and according to the cloud point. The casting solution flow rate, bore fluid rate, and spinning rate were constant. The details of the spinning process were reported elsewhere [26,27]. It is well known, membrane morphology and performance are greatly affected by the pre-treatment or drying procedure. The effect of membrane pre-treatment and temperature on the membrane structure and separation performance was in detail by Jonathan et al. [34,35,36]. In this study, the prepared membranes were immersed in deionized water for three days to extract residual DMAc and PEG200 in the membrane. The deionized water was renewed every day. Subsequently, hollow fiber membranes were immersed in 20 wt.% aqueous glycerol solution for three days and dried at room temperature, to obtain dry hollow fiber membranes for testing.

### 2.5. Characterization of the PES Membranes

#### 2.5.1. Morphology

The cross sections and surface morphologies of the hollow fiber membranes were observed by scanning electron microscopy (SEM) (S-3400Ⅱ, Hitachi High-Technologies, Tokyo, Japan). The cross-sections of the dry membranes were fractured in liquid nitrogen, and then sputtered with gold under vacuum. The diameters of the prepared membranes were measured by an optical microscope.

#### 2.5.2. Permeation Performance

First, the modules were immersed in deionized water for eliminating glycerol in the prepared membranes. BSA aqueous solution was used as the feed solution; the concentration of BSA was 300 ppm. A self-designed measuring device was reported in the previous article [37]. All the permeation tests were conducted out at room temperature with a constant feed pressure of 0.1 MPa. Second, the immersed modules were pre-pressured at 0.1 MPa with pure water for 0.5 h before test. After that the pure water permeation flux (J_w_) and the rejection rate (R) of BSA aqueous solution were determined. The BSA concentrations of the feed and the permeate solutions were analyzed by UV-300 spectrophotometer (Shimadzu, Kyoto, Japan). Each sample was tested three times and averaged. The J_w_ and the R are defined as Equations (1) and (2), respectively [25]:

(1) $$J_{w}=\frac{V}{A\times t}$$

(2) $$R=(1-\frac{C_{P}}{C_{F}})\times 100\%$$

where J_w_ is the deionized water permeation flux (L·m^−2^·h^−1^), A is the effective area of the prepared membrane, V is volume of the permeate pure water (L), t is the permeation time (h). R is the rejection rate of BSA (%), CP and CF are the BSA concentrations of the feed and the permeate solution (wt. %), respectively.

#### 2.5.3. Porosity and Pore Size

Membrane porosity ε (%) is determined by the dry-wet weight method [38] using the equation as follows:

(3) $$\varepsilon =\frac{{(m}_{w}-m_{d}{)/\rho }_{\mathrm{water}}}{{(m}_{w}-m_{d}{)/\rho }_{\mathrm{water}}{+m}_{d}{/\rho }_{p}}$$

where m_w_, m_d_, ρ_water_ and ρ_p_ are the wet membrane weight (g), dry membrane weight (g), the density of deionised water and PES (1.370 g·cm^−3^), respectively.

Mean pore size (r_m_) was determined by the filtration velocity method and described by the Guerout–Elford–Ferry equation [39]:

(4) $$r_{m}=\sqrt{\frac{\left(2.9-1.75\varepsilon \right)\times 8\eta \mathrm{hQ}}{\varepsilon \times A\times \Delta p}}$$

where η is the viscosity of water (8.9 × 10^−4^ Pa·s), h is membrane thickness (mm), Q is deionized water flux (mL·s^−1^), ε is membrane porosity, A is the effective area of the membrane and ΔP is the trans-membrane pressure (0.1 MPa).

#### 2.5.4. Hydrophilicity

The hydrophilicity of prepared membranes was characterized by the static pure water contact angle (θ) of membrane outer surface. A JC2000A contact angle meter (Zhongchen Digital Equipment Co. Ltd., Shanghai, China) was used to investigate the θ of the membranes. The volume of the water droplet was 0.2 μL. When water droplet dispersed on the membrane surface, the camera enabling image captured. The θ was analyzed through the calculated software from the image. Each sample was tested five times and averaged.

#### 2.5.5. EDX

The Energy Dispersive X-ray (EDX) spectrometer (Falion 60S, EDAX Inc., Berwyn, PA, USA) was used to investigate the element composition on the outer surface of the prepared membranes.

#### 2.5.6. Thermal Stability

The membrane thermal stability was investigated via thermogravimetric analysis to determine and compare the effect of SPES on the thermal stability of membrane. All tests were performed from 50 to 900 °C at a heating rate of 10°C·min^−1^ and a nitrogen atmosphere on a thermogravimetric analyzer Discovery series (Discovery TGA, TA instruments, New Castle, DE, USA). The onset decomposition temperature and peak decomposition temperature were taken as T_d_^on^ and T_d_^peak^, respectively. 

#### 2.5.7. Mechanical Properties

The tensile strength, Young’s modulus and elongation at break of the prepared membranes were measured by a tensile testing apparatus (QJ-210A, Qingji Instrumentation Science and Technology Co. Ltd., Shanghai, China). The loading speed was 50 mm·min^−1^, and the distance between gauges was 50 mm. Each membrane sample was subjected to 5 times tensile tests and averaged. 

## 3. Results and Discussion

### 3.1. Cloud Point

**Figure 1:**
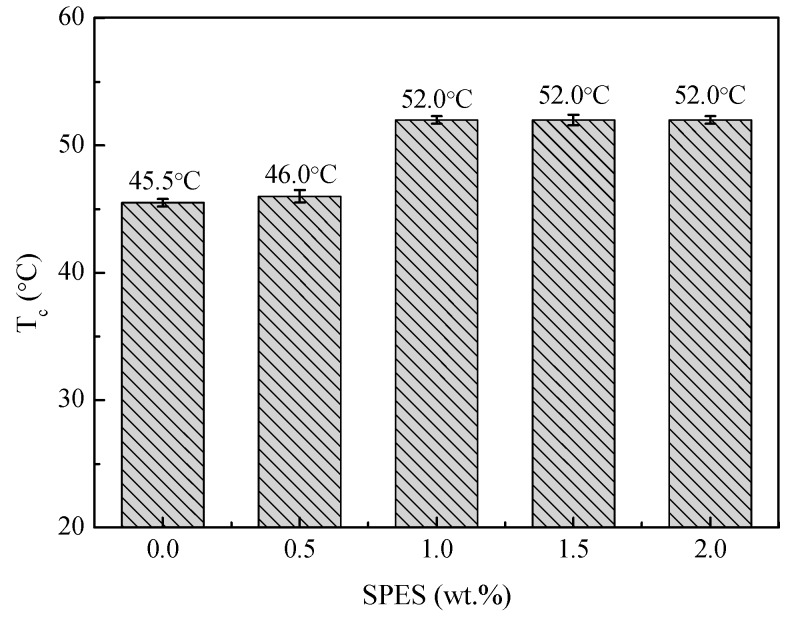
Cloud points of different PES/SPES/DMAc/PEG200 casting solutions.

The effects of SPES content on phase separation temperature are shown in Figure 1. First, for the casting solution MPESS-1, the cloud point was slightly higher than that of MPESS-0 system. The reason for this is the intermolecular hydrogen-bonding interactions between PES/SPES and mixed solvent (DMAc/PEG200). On the account of LCST casting solution, a small addition of SPES does not have a significant effect on the cloud point. 

Second, the cloud point was shifted to higher temperatures as SPES concentration increased to 1 wt.%. The result can be explained by increasing hydrogen-bonding interactions between PES/SPES and mixed solvent with an increase in the hydrophilic sulfonic group of the casting solution. When the concentration of SPES increases, the interactions between PES/SPES and the mixed solvent become stronger, and consequently LCST casting solution (MPESS-1) induces phase separation of at high temperature.

Third, the cloud point showed similar values when the concentration of SPES continued to increase. This indicates that the increase of SPES concentration is not attributed to the hydrogen-bonding interactions when the concentration of SPES is higher than 1 wt.%. The reason for this is related with the hydrogen-bonding interactions. Due to good compatibility between PES/SPES and DMAc, PES and SPES can dissolve in the mixed solvent (DMAc/PEG200) and keep stability at room temperature (in spite of incompatibility between PES/SPES and PEG200). When the content of SPES is 1.0 wt.%, the hydrogen-bonding reactions between the mixed solvent (DMAc/PEG200) and the hydrophilic sulfonic groups are saturated. When the content of SPES continues to increase, no more hydrogen-bonding interactions are formed, in spite of the increase of sulfonic groups. Correspondingly, the cloud point is almost constant.

### 3.2. Viscosity

**Figure 2:**
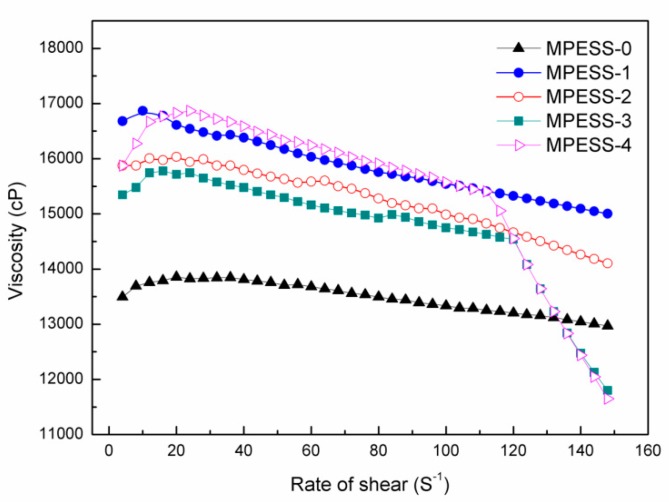
Shear viscosities of the PES/SPES/DMAc/PEG200 casting solutions.

The shear viscosities as a function of shear rate for the PES/SPES/DMAc/PEG200 casting solutions with different contents of SPES are illustrated in Figure 2. First, with the addition of SPES, the initial viscosities of PES/SPES/DMAc/PEG200 casting solutions are higher than that of the pure PES casting solution (MPESS-0). The result indicates that SPES molecules entangle itself with PES molecules in the casting solution, which leads to the increase of initial viscosities. Second, when the SPES content is 1.0 wt.% and 1.5 wt.%, the viscosity of MPESS-2 and MPESS-3 decreased. A possible reason is that the hydrophilic sulfonic groups (-SO_3_H) are over-saturated, which results in an increase of extension of SPES molecule chain (due to repulsion between -SO_3_H groups of SPES). The over-saturated part of SPES serves as a lubricant, which leads to a decrease of viscosity. When the SPES content is 2.0 wt.%, the over-saturated content of SPES increases. The entanglement and hydrogen bonding is stronger than lubrication effect, which leads to the increase of viscosity. Third, the casting solutions exhibit a shear thinning phenomenon, and the shear thinning phenomenon becomes more obvious with the increase of SPES content. Especially, when the SPES content is 1.5 wt.% and 2.0%, the viscosities of MPESS-3 and MPESS-4 are less than the pure PES casting solutions at high shear rate. It indicates that the over-saturated SPES molecules are relative extend. Correspondingly, as the shear rate increases the entanglement between hydrophobic PES molecules and hydrophilic SPES molecules are easily destroyed due to repulsion among over-saturated sulfonic groups. 

### 3.3. Light Transmittance Measurement

**Figure 3:**
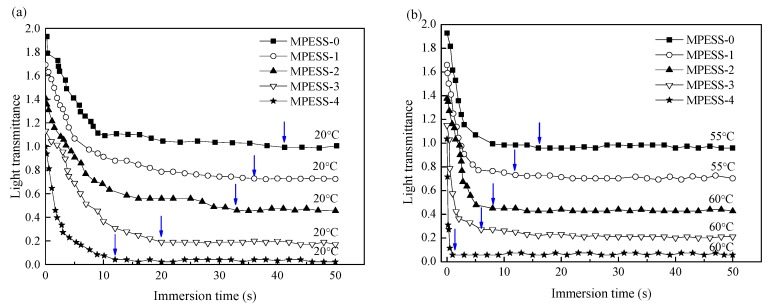
Light transmittance curve obtained by (**a**) NIPS and (**b**) Reverse Thermally Induced Phase Separation (RTIPS) mechanism. (Profiles are shifted for the purpose of clarity).

To follow the difference between NIPS and non-solvent assisted RTIPS membrane formation process, light transmittance tests were carried out. As shown in Figure 3a, when the coagulation bath temperature was 20 °C which is lower than the cloud point, the phase separation process is NIPS. The light transmittance of all the casting solutions decreases quickly and then changes slowly until unalterable in the end. With the increase of SPES content, the descending rate increased and the whole phase separation time reduced from 40 to 12 s. This happens since SPES was used as a hydrophilic additive in the casting solutions, and the membrane formation process is instantaneous demixing of NIPS process (mass transfer between the casting solution and the coagulation bath), which increases the phase separation rate.

As shown in Figure 3b, when the coagulation bath temperature is higher than the cloud point, the dominant process is non-solvent assisted RTIPS. The light transmittance of all the casting solutions decreases more quickly at first than that of casting solutions in Figure 3a. With the increase of SPES content, the descending rate increased and the whole phase separation time reduced from 16 to 2 s. It indicates that RTIPS is the dominating process and confirmed that heat transfer rate is much faster than mass transfer rate [25].

### 3.4. Membrane Morphology

**Figure 4:**
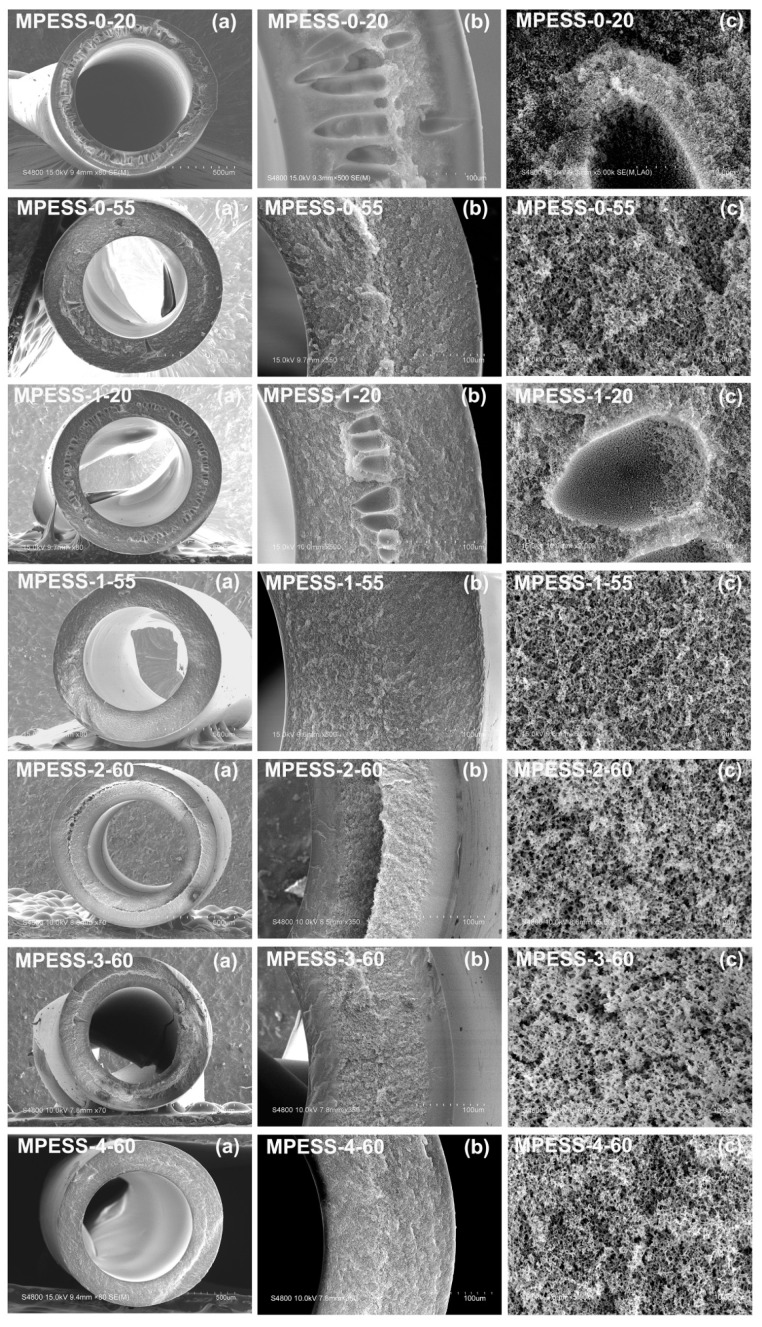
SEM micrographs of the PES hollow fiber membranes. (**a**) full cross-section; (**b**) part of cross-section; (**c**) enlarged cross-section.

**Figure 5:**
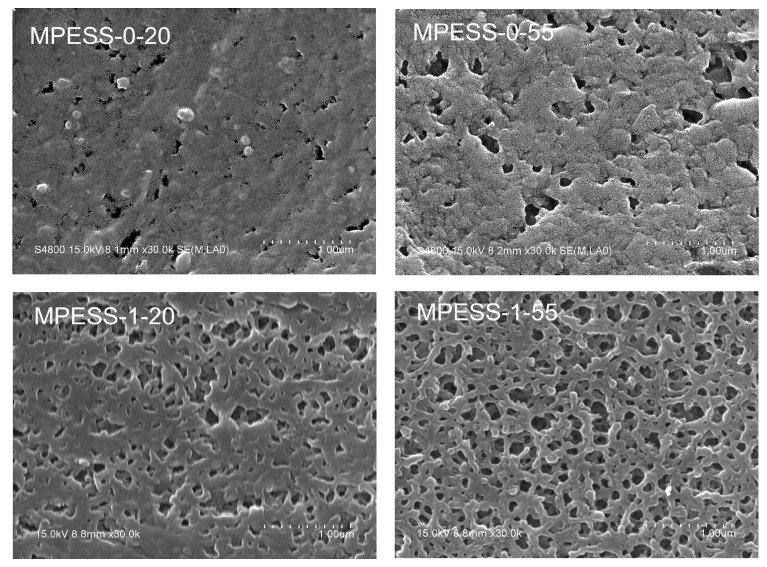
SEM micrographs of the outer surfaces of PES hollow fiber membranes.

The SEM micrographs of PES hollow fiber membranes with different contents of SPES, prepared by the NIPS and non-solvent assisted RTIPS processes, are shown in Figure 4 and Figure 5. The cloud point of the casting solution MPESS-0 is 45.5 °C. When the coagulation bath temperature was 20 °C, which was lower than the T_c_, the major driving force of membrane formation is mass transfer. The membrane MPESS-0-20, with dense skin layers and finger-like pores, was formed in the cross-section as shown in Figure 4. When the coagulation bath temperature was 55 °C, which is higher than the T_c_, the PES membrane formation was dominated by the non-solvent assisted RTIPS process. Membrane MPESS-0-55 with a bi-continuous structure was obtained, as shown in Figure 4, which is the representative morphology of membranes with high flux and good mechnical properties. From the SEM micrographs in Figure 4, it can be seen that the MPESS-1-20 and MPESS-1-55 membranes have similar morphology to the MPESS-0-20 and MPESS-0-55 membranes, respectivlely; however, the dense skin layers of the membrane MPESS-1-20 change to become thinner than that of the membrane MPESS-0-20 due to increase of viscosity of the casting solution MPESS-1. These observations indicate that the dominant membrane formation process is not changed by the addition of SPES. Correspondingly, a dense outer surface was found in the membrane MPESS-0-20, as shown in Figure 5, which is due to instantaneous phase separation of the NIPS process. The membrane MPESS-1-20 with porous outer surfaces resulted from the addition of hydrophilic SPES, which leads to the increase of viscosity of casting solution, as shown in Figure 2 and Figure 5. Meanwhile, the membrane MPESS-0-55 with porous outer surface resulted from non-solvent assisted RTIPS mechanism. Under the combined effect of addition of hydrophilic SPES and RTIPS membrane forming mechanism, the outer surface of membrane MPESS-1-55 presents a more porous structure with interconnected. Comparing to the pure PES membrane (MPESS-0-55), the pore connectivity is better in the membrane MPESS-1-55. This indicates that the degree of porosity of the membrane surface is improved due to the presence of hydrophilic sulfonic groups, which increases the membrane flux.

SEM images of PES/SPES membranes prepared with different SPES contents via the non-solvent assisted RTIPS method are shown in Figure 4. As shown in Figure 1, the cloud point of the casting solutions with different SPES contents is between 45.5 °C and 52.0 °C. When the membrane-forming temperature is 55 °C or 60 °C, which is higher than the T_c_, the membrane formation process is dominated by the non-solvent assisted RTIPS mechanism, due to the major driving force of phase separation being heat transfer instead of mass transfer. All the membranes (MPESS-0-55, MPESS-1-55, MPESS-2-60, MPESS-3-60 and MPESS-4-60) were formed with a bi-continuous structure in the cross section, as shown in Figure 4, which demonstrates the membrane formation process is non-solvent assisted RTIPS mechanism [25,30].

In summary, based on the SEM image results, it is evident that an optimization of membrane structure has been relized by adding hydrophilic SPES.

### 3.5. Permeation Properties, Porosity and Pore Size

**Figure 6:**
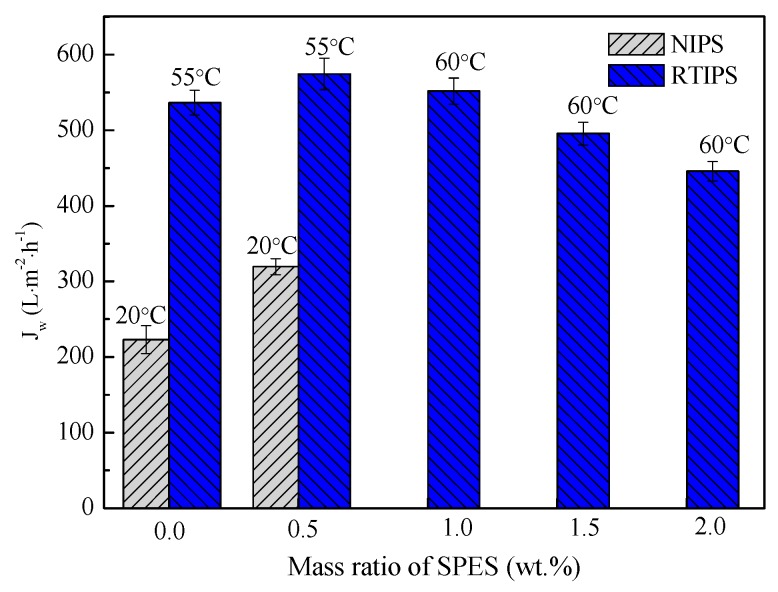
Pure water permeation flux of the prepared PES hollow fiber membranes.

**Figure 7:**
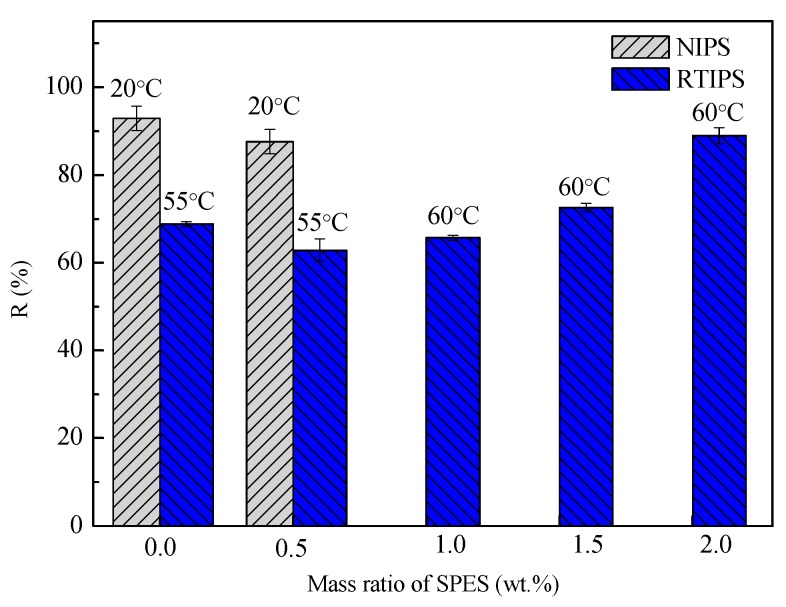
Rejection rate of the prepared PES hollow fiber membranes.

**Figure 8:**
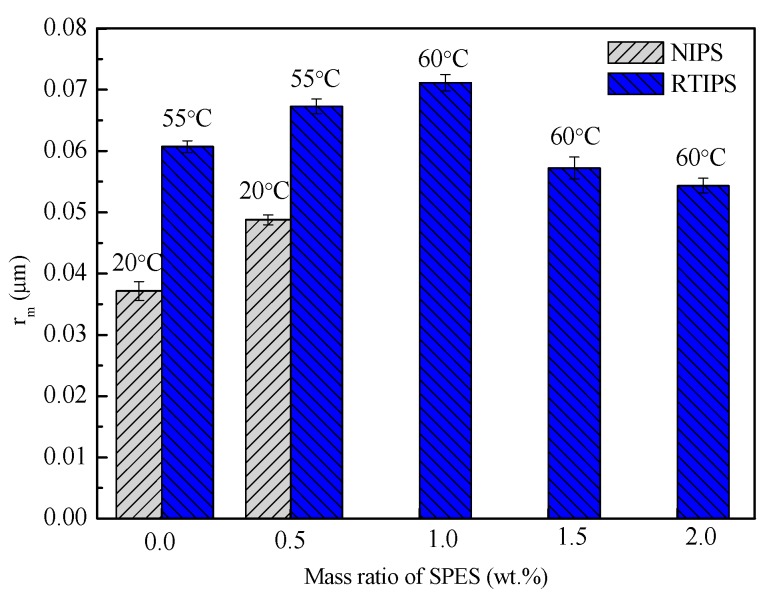
Mean pore size of the prepared PES hollow fiber membranes.

**Figure 9:**
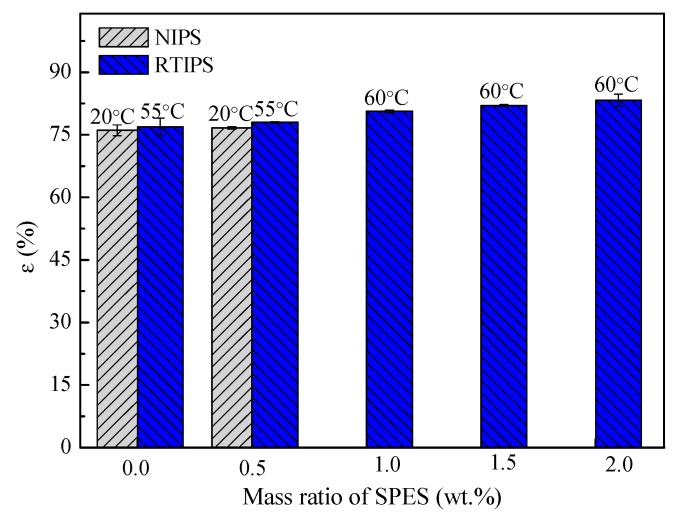
Porosity of the prepared PES hollow fiber membranes.

The pure water permeation flux, rejection rate, porosity, and mean pore size of PES hollow fiber membranes prepared with different SPES content and coagulation bath temperature, are shown in Figure 6, Figure 7, Figure 8 and Figure 9. First, for MPESS-0-55 and MPESS-1-55, the membrane formation mechanism is controlled by the non-solvent assisted RTIPS mechanism, and J_w_ is higher than that of the corresponding membranes (MPESS-0-20 and MPESS-1-20) prepared by the NIPS mechanism (see Figure 6). As shown in Figure 8, the MPESS-0-55 and MPESS-1-55 have a bigger r_m_ than that of the MPESS-0-20 and MPESS-1-20, so the rejection rate of the membrane MPESS-0-55 and the MPESS-1-55 has a lower value (see Figure 8). 

Second, the J_w_ of the PES membranes prepared by the non-solvent assisted RTIPS reaches maximum value for the MPESS-1-55 membrane following a decrease in SPES content (see Figure 6) Thus, the rejection rate of the corresponding membranes presents a decrease with an increase in SPES contents in the following descending order: MPESS-2-60, MPESS-3-60 and MPESS-4-60 (see Figure 7). The r_m_ of membranes prepared via the non-solvent assisted RTIPS process reaches a peak value for the membrane MPESS-2-60, as shown in Figure 8, and then the r_m_ present a downtrend with an increase in SPES contents. 

Third, although from the results in Figure 9 it can be seen clearly that there is little difference in porosity (ε), which is due to the same polymer content, but the porosity of the membranes prepared via non-solvent assisted RTIPS process increases in line with a slightly increase in SPES concentration. This is due to the hydrophilicity of SPES molecules accelerates the diffusion of pure water from the coagulation bath to the casting solution during the spinning process, which results in the slightly increase of the porosity. 

Based on these results, it can be seen clearly that PES/SPES hollow fiber membranes prepared by the non-solvent assisted RTIPS process can be applied as a new method for preparing hydrophilic membranes with high flux. 

### 3.6. Hydrophilicity and EDX Analysis

**Figure 10:**
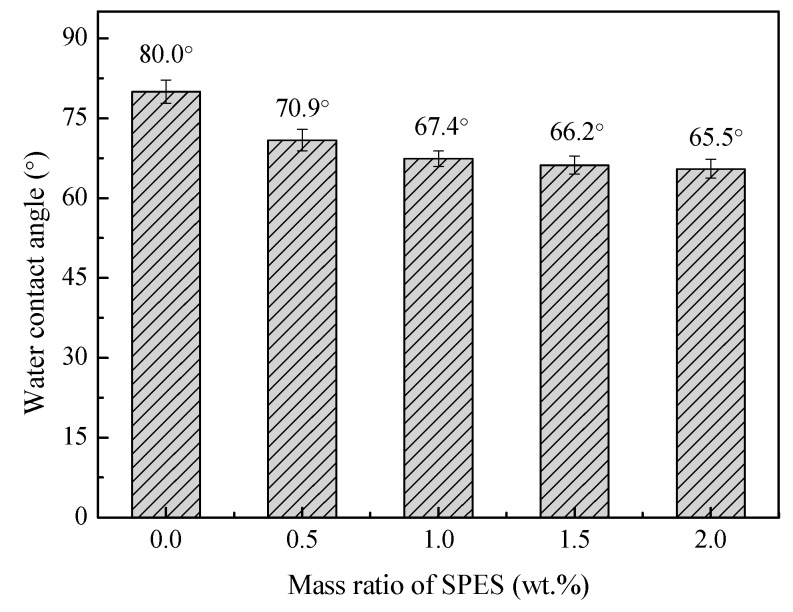
The static pure water contact angles of the prepared membranes via non-solvent assisted RTIPS.

**Figure 11:**
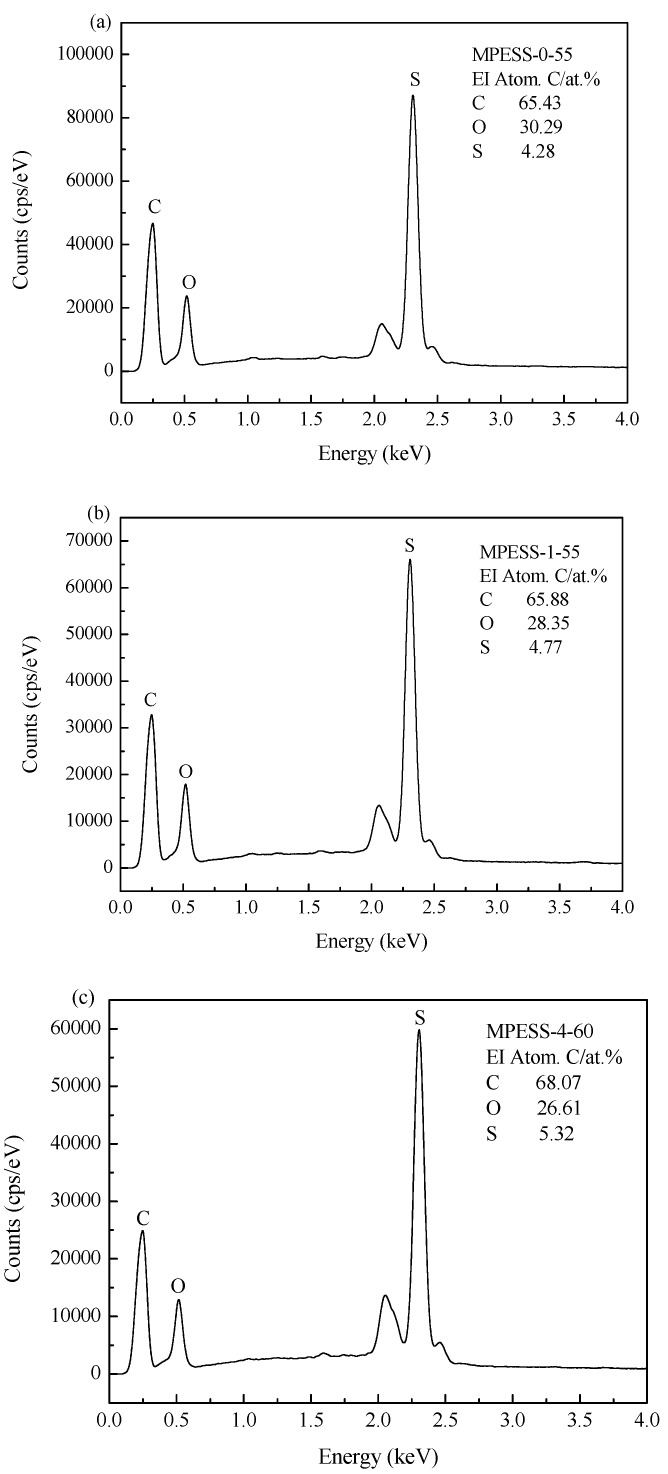
The EDX spectra of the prepared PES hollow fiber membranes.

The static pure water contact angles (θ) of the prepared PES hollow fiber membranes are shown in Figure 10. The θ for the pure PES hollow fiber membrane MPESS-0-55 is 80.0°. When the SPES content increased from 0 to 2.0 wt.%, θ decreased from 80.0° to 65.5°. Since SPES is hydrophilic and improve the hydrophilicity of the PES membrane. The existence of the SPES molecules is verified by the EDX spectra of the outer surface of the prepared hollow fiber membranes, as shown in Figure 11. The C/S ratios of the MPESS-0-55, MPESS-1-55 and the MPESS-4-60 are 15.29, 14.03 and 12.79, respectively. This is due to that the relative content of sulphur in SPES molecules is high; the decrease of the C/S ratio shows the increase of the SPES amount on the membrane outer surface, which verifies the decline tendency of contact angles. 

### 3.7. Thermal Stability

**Figure 12:**
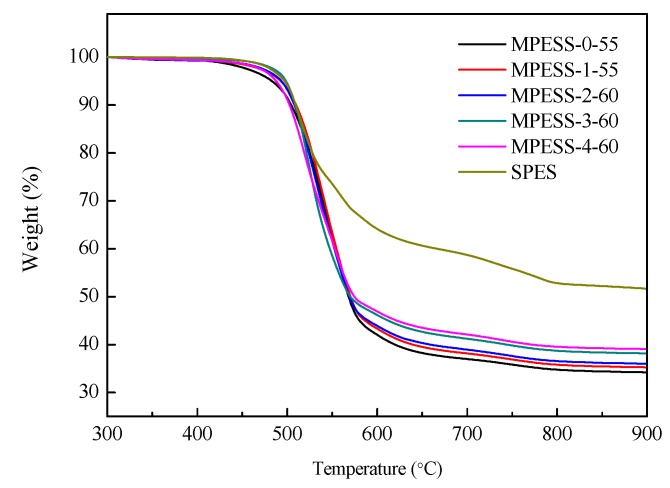
TGA curves of the prepared PES hollow fiber membranes.

Thermal stability properties of the prepared hollow fiber membranes are illustrated in Figure 12, and the thermal decomposition temperatures T_d_^on^ and T_d_^peak^ are listed in Table 3. It could be seen that the thermal stability of the prepared PES/SPES membranes is between that of the pure PES and pure SPES. With the increase of SPES content, the thermal decomposition temperature T_d_^on^ and T_d_^peak^ decreased. Still, for the prepared PES/SPES hollow fiber membranes, it has good thermal stability because the T_d_^on^ and T_d_^peak^ are higher than 490 °C and 516 °C, respectively, which are significantly higher than the conventional use temperature of membrane. 

### 3.8. Mechanical Properties

**Figure 13:**
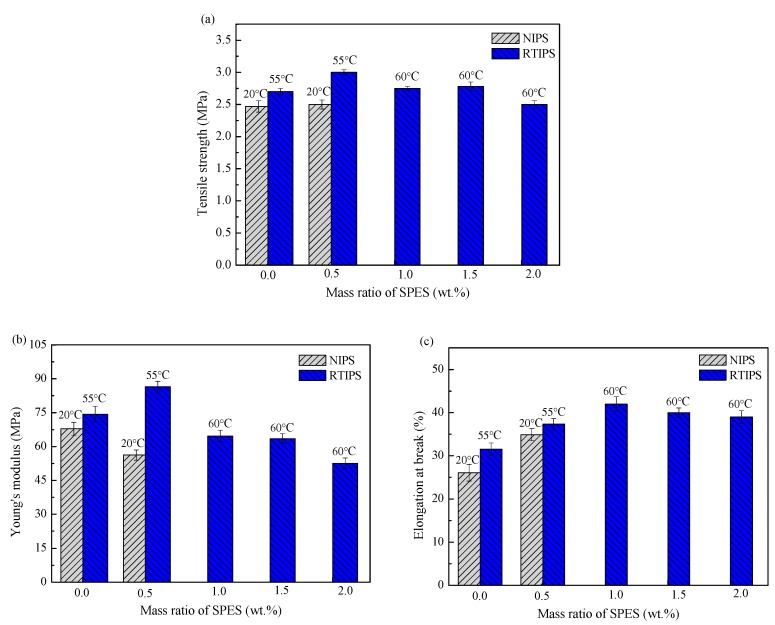
Mechanical properties of PES membranes.

The mechanical properties of PES hollow fiber membranes prepared with different SPES content and coagulation bath temperatures are shown in Figure 13. For MPESS-0-20 and MPESS-1-20, the membrane-forming mechanism is the NIPS process, in which the tensile strength (σ_t_), the Young’s modulus (E_t_) and elongation at break (ε_t_) are lower than for the corresponding membranes (MPESS-0-55 and MPESS-1-55) prepared by the non-solvent assisted RTIPS mechanism (see Figure 13a–c). As shown in Figure 4, the finger-like pore structure on membrane cross-section disappears and a bi-continuous structure is formed when the coagulation bath temperature is higher than the T_c,_, which results in an improvement to the tensile properties of the membrane.

As Figure 13b shows, the tensile strength and the Young’s modulus of the PES hollow fiber membranes prepared with the non-solvent assisted RTIPS method reach the maximum values at 0.5 wt.% SPES content. The membrane structure becomes heterogeneous bi-continuous following an increase in SPES content, which results in an decrease in tensile strength and Young’s modulus. The elongation at break reached its peak values at 1 wt.% SPES content. Furthermore, Figure 13c shows that the elongation at break of the membranes MPESS-1-55, MPESS-2-60, MPESS-3-60 and MPESS-4-60 are higher than that of the pure PES membrane. This indicates that the toughness of the PES membrane was enhanced by the addition of SPES. 

## 4. Conclusions

Hydrophilic PES hollow fiber membranes were prepared via an non-solvent assisted RTIPS process under the addition of SPES. With regard to casting solutions, DMAc was used as the good solvent, while PEG200 was used as the non-solvent. The PES/SPES/DMAc/PEG200 casting solution was LCST systems, and the cloud point increased with the increase in SPES content. When the SPEG content increases, the initial viscosities of the casting solutions increased, but the casting solutions exhibited a shear thinning phenomenon. When the membrane formation temperature was higher than the cloud point, the membrane-forming dominant process was non-solvent assisted RTIPS process. The phase separation rates of non-solvent assisted RTIPS process are faster than that of NIPS process, and the phase separation rate increased with the increase of SPES content.

On the one hand, when the membrane-forming mechanism was the NIPS process, a finger-like pore morphology was present in the membrane. On the other hand, when the membrane-forming mechanism was mainly controlled by the non-solvent assisted RTIPS process, a bi-continuous morphology was formed in the membranes. Comparing the pure PES membrane, a more porous surface structure was obtained with the addition of SPES, which would help to increase membrane flux. The membranes prepared by applying the non-solvent assisted RTIPS process present higher pure water flux than that of the membranes prepared with the NIPS process, and the pure water flux for the membranes prepared by the RTIPS reaches peak value when the SPES content is 0.5 wt.%. The porosity of the membranes prepared by the non-solvent assisted RTIPS process slightly increased with an increase in SPES content, while the pure water contact angle and the thermal decomposition temperature of membranes prepared by the non-solvent assisted RTIPS method decreased with an increase in SPES content. The advantages of the addition of SPES are reflected by the relatively high flux and high hydrophilicity at 0.5–1.0 wt.% SPES content. 

When the membrane-forming process was the non-solvent assisted RTIPS, the tensile strength, the Young’s modulus and the elongation at break were higher than of the corresponding membranes prepared by the NIPS mechanism. With the addition of SPES, membrane toughness improved; while the tensile strength and Young’s modulus reached peak value at 0.5 wt.% SPES content. The preferred content of SPES is 0.5 wt.%. We therefore conclude that the advantage of the SPES additive is the easy preparation of membranes with good hydrophilicity and more porous surface morphology resulting in good permeation properties. 

## Figures and Tables

**Table 1:** Compositions of casting solutions.

Casting Solutions	Casting Solution Compositions (wt.%)			
	PES	SPES	DMAc	PEG200
MPESS-0	17.0	0	20.75	62.25
MPESS-1	16.5	0.5	20.75	62.25
MPESS-2	16.0	1.0	20.75	62.25
MPESS-3	15.5	1.5	20.75	62.25
MPESS-4	15.0	2.0	20.75	62.25

**Table 2:** Temperature of the membrane formation.

Membranes	Internal and External Bath Temperature (°C)
MPESS-0-20	20
MPESS-0-55	55
MPESS-1-20	20
MPESS-1-55	55
MPESS-2-60	60
MPESS-3-60	60
MPESS-4-60	60

**Table 3:** Thermal decomposition temperature of the prepared PES/SPES membranes.

Membranes	MPESS-0-55	MPESS-1-55	MPESS-2-60	MPESS-3-60	MPESS-4-60	SPES
T_d_^on^ (°C)	500.8	500.0	496.9	497.1	490.3	492.1
T_d_^peak^ (°C)	551.2	539.0	528.0	523.6	516.1	514.7
